# The Promise and Challenge of Digital Biology

**DOI:** 10.4172/2155-9538.1000e118

**Published:** 2013-12-04

**Authors:** Mark E Minie, Ram Samudrala

**Affiliations:** 1 Bioengineering Department, University of Washington, USA; 2 Microbiology Department, University of Washington, USA

## Introduction

**Figure 1:**
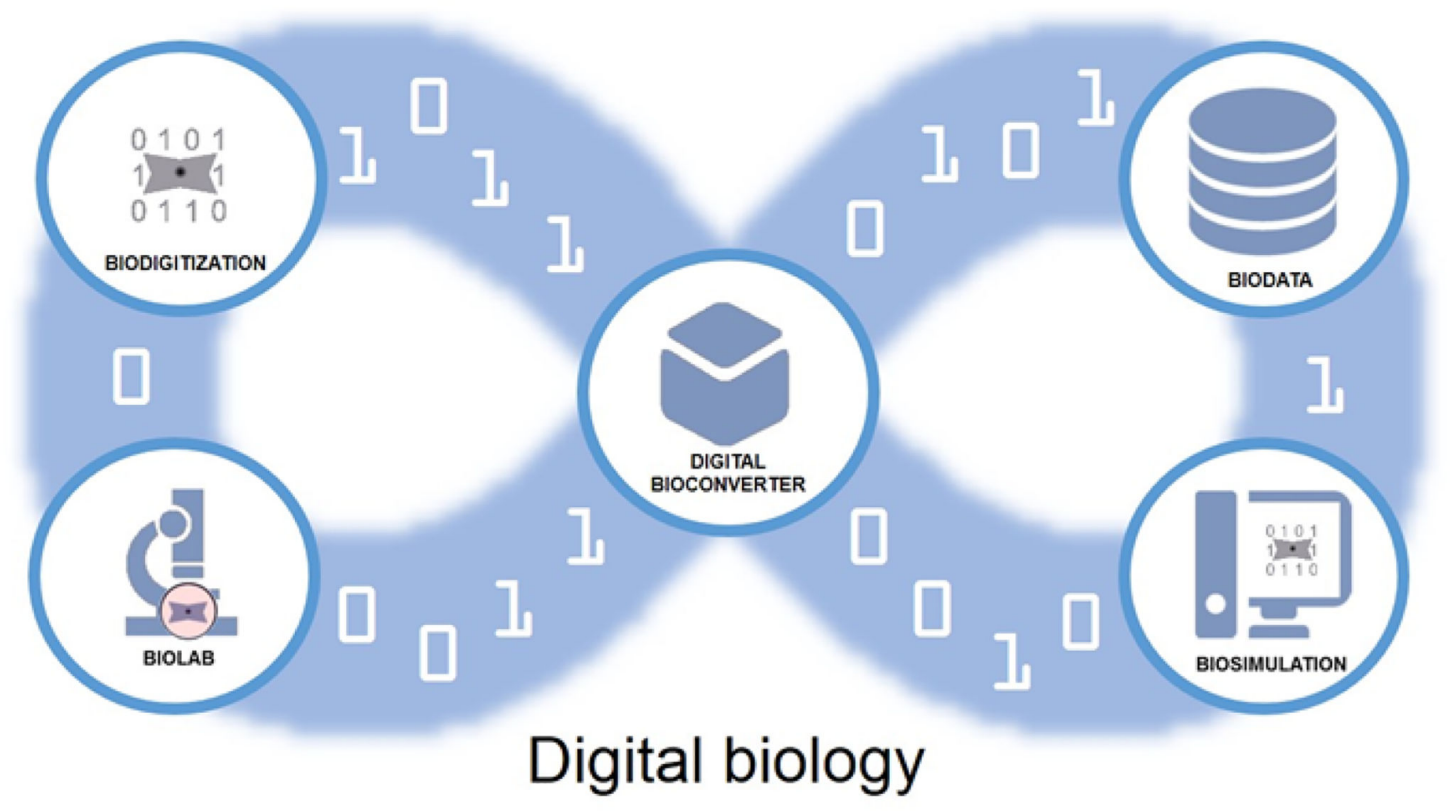
The digital biology loop, with the digital bioconverter, a tool for instantiating data driven biosimulations into biomolecules and cells for analysis at the lab bench, digitization and further simulation and analysis.

The era of Digital Biology began in 2010 with the “rebooting” of a bacterial cell using a synthetic DNA genome created from a digital template stored on a computer [1]. With this event, the creation of *Mycoplasma laboratorium* (nicknamed “Synthea”), came the first complete proof that DNA was the true software of life. Cells could be simulated digitally and the simulations could be tested against reality by reprograming cytoplasm with synthetic genomes generated from the digital DNA sequences driving those simulations. This in turn has created the expectation and promise that a deeper understanding of cellular function and thus life itself could be achieved on an infinite iterative loop of computer modeling and chemical synthesis (Figure 1) [2].

Key components of the digital biology loop are 1) a detailed digital mapping of living systems and their biomolecular parts and the interactions of such parts-biodigitization, 2) accessible databases containing/managing this biodata, 3) computer simulation algorithms of cells driven by digital DNA sequences encoding the biomolecular parts and interactions-biosimulation, 4) laboratory technologies to deeply analyze the resulting synthetic cells-biolab-and finally and centrally 5) the digital biological converter (digital bioconverter for short). In these early days of digital biology, each of these components presents exciting bioengineering, bioscience and biomedical challenges.

## Biodigitization and Biodata

**Figure 2:**
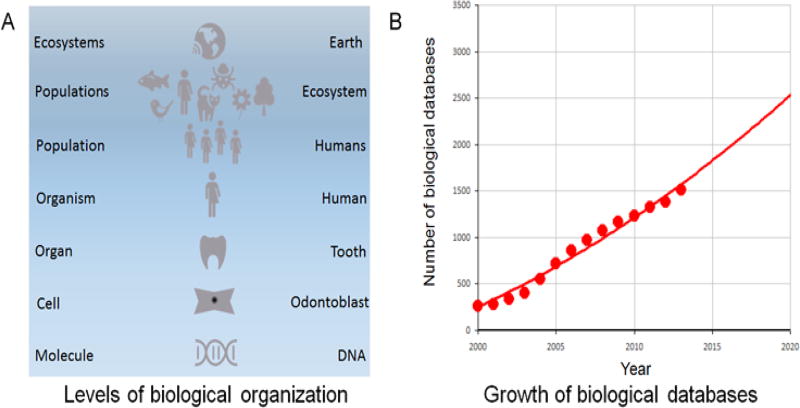
A. Biological data is being acquired at every level of Earth’s biosphere and B. the digitized biodata is being incorporated into Web accessible databases at a doubling rate of once every 5 years (from [6]).

Every aspect of Earth’s biosphere is currently being digitized, from the molecular to the planetary levels and the data entered into an ever-growing collection of biologically oriented Internet databases-a mirror backup image of terrestrial life is literally being created (Figure2A)[3]. The digital acquisition of DNA sequences of phage, viruses, bacteria and human cells, the 3D structures of biomolecules as well as the detailed cellular structures and tissue and organ architecture has been underway in earnest for more than two decades now. With the advent of molecular imaging, electronic medical records, and “Big Data” [4,5], every aspect of individual organisms, populations, and ecosystems are now also being fed into the Internet based DataStream. Online biological databases are also on track for doubling every 5 years [3,6] (Figure 2B). The sheer volume of such data now threatens to overtake current data storage and search technology, and may require the development of novel technologies, including nucleic acid based data storage [7] and quantum computing [8].

## Biosimulation and Biolab

**Figure 3:**
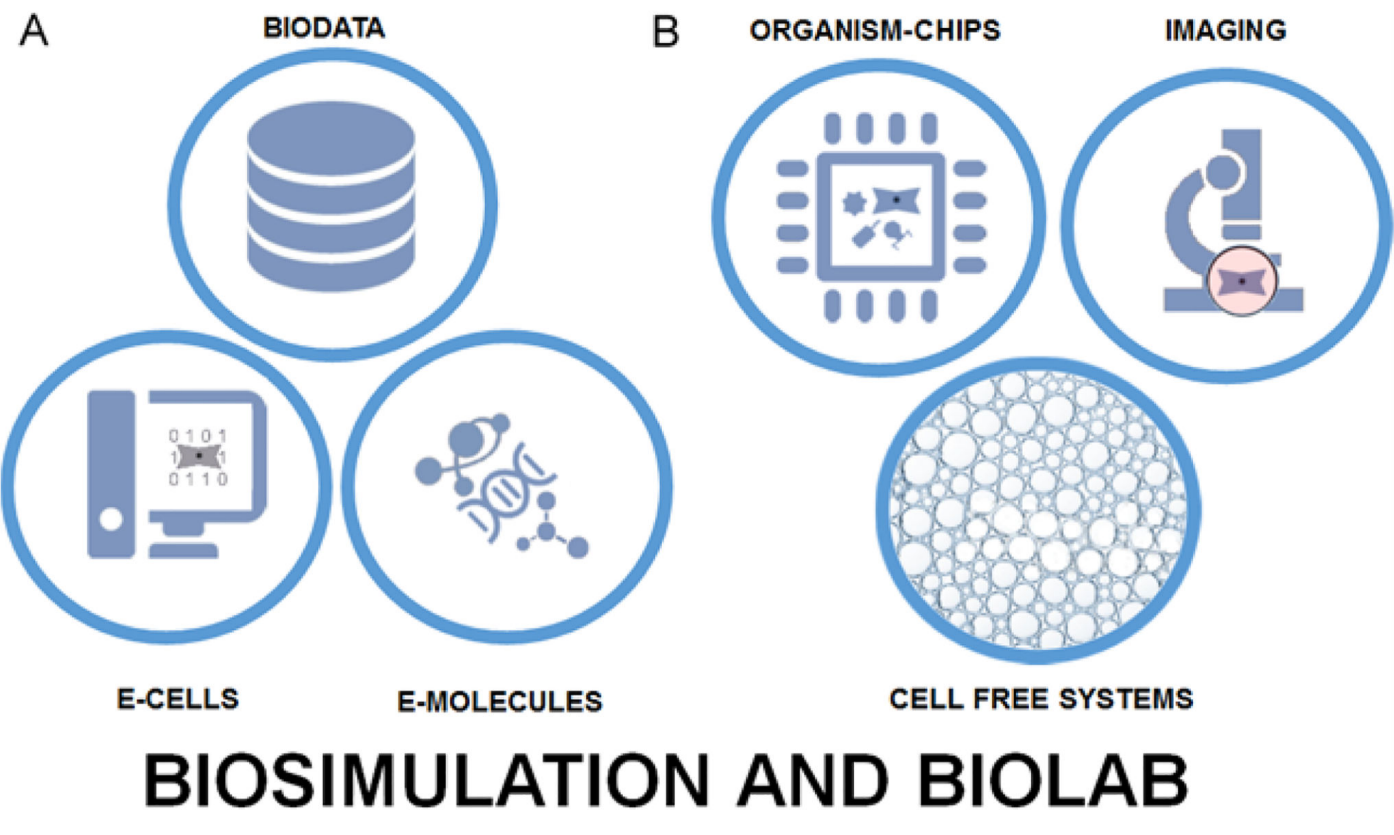
A. Components for biosimulation-digitized biodata, digital electronic cell models (E-CELLS), and digital electronic molecular models (E-MOLECULES). B. Biolab tools for analysis-novel cell/viruses on chip systems (ORGANISM-CHIPS), advanced microscopy (IMAGING) and advanced *in vitro* biochemistry (CELL FREE SYSTEMS).

While a number of simulations of cells have been developed over the last two decades [9–11], so far only one specifically driven by a digitized DNA genome from a real cell [12], Mycoplasma genitalium, has been published. The advent of synthetic biology tools such as Tinkercell [12–14] and database driven animation [15] provide strong starts for the tools that will be needed. Detailed electronic images of biomolecules useful in simulations are already readily available from such sources as PubChem and Biosystems [6]. On the other side of the issue, biolab tools for analyzing and manipulating synthetically produced living cells are arising with breathtaking speed. Molecular imaging of structures within living cells is now possible [16–19], and even the direct physical manipulation of cellular components using optical tweezers is a routine technique [20]. Additive manufacturing tools now make the construction of artificial biofilms and organs for research a reality [21–28] (Figure 3).

## Digital Bioconverter

**Figure 4:**
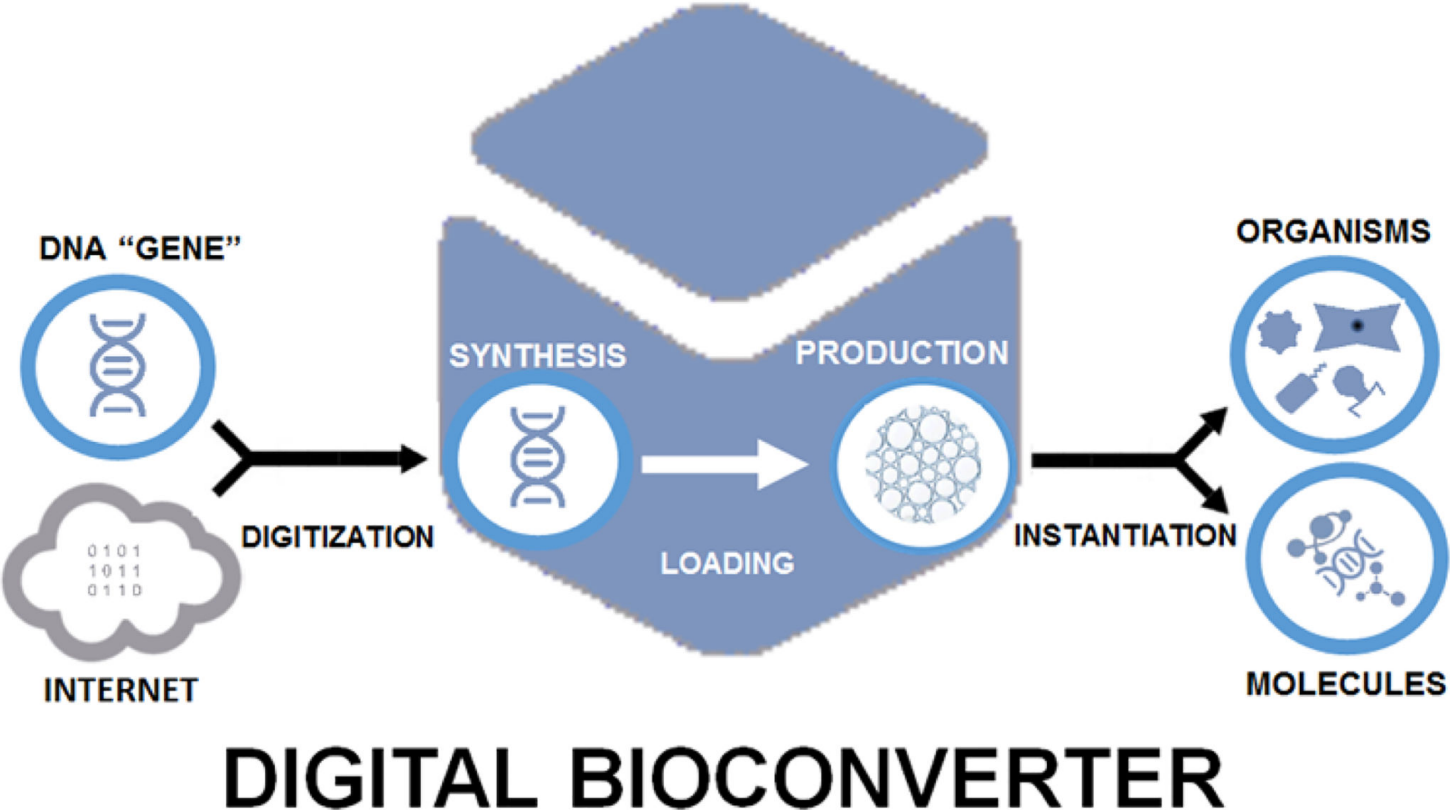
The digital bioconverter, conveying electronic digitized information to the biological realm *via* DNA.

The key “gadget” in digital biology, the digital bioconverter (Figure 4), currently exists as a prototype [2] and will likely eventually evolve into a miniaturized commercially produced laboratory instrument. Such a system would allow the convenient production of cells, viruses and biological molecules directly from digitized gene encoding DNA sequences, and eventually could be as central to basic bioscience research as automated DNA sequencers are today. Significant challenges must be met before this is realized, however. While synthesis of large genomes is now possible, it remains complex and expensive. An alternative to cellular transformation could be realized *via* cell free systems-DNA could be loaded into such systems and then drive the production of biomolecules or organisms through a further instantiation step-possibly through reconstitution of cells and viruses (organisms) from *in vitro* systems [29–32] or the cell free synthesis of biomolecules.

## Grand Synthesis

Increasingly available digitized biodata coupled with advanced biosynthetic synthesis are leading to a new era of biology where electronic digital simulations can be converted to cells and biological molecules-the era of digital biology. While several major engineering and computing challenges must be tackled, these are not insurmountable and are the objects of vigorous technological innovation. In particular, the development of a standardized and commercially available bioconversion device will be critical, and with such a device in eventual widespread use a rapid cycle of model driven understanding of biological systems will get underway. Such a device and the concept of digital biology will have applications in many fields, including astrobiology [33–35], medicine [36–38], nanotechnology [39], bioinformatics [36,37,40–47], drug repurposing [48] and pharmacoengineering [49–51], while presenting the promise of placing bioengineering and biomedicine on a Moore’s Law-like curve of exponentially increasing understanding and providing exquisite control of living systems.
